# Melanotrichoblastoma: sixth case report in the literature^☆^

**DOI:** 10.1016/j.abd.2022.07.011

**Published:** 2023-06-29

**Authors:** Juliana Polizel Ocanha-Xavier, José Cândido Caldeira Xavier-Júnior

**Affiliations:** a Department of Dermatology, Private Practice, Araçatuba, SP, Brazil; b Department of Pathology, Faculty of Medicine, Universidade do Estado de São Paulo, Botucatu, SP, Brazil; a Department of Pathology, Faculty of Medicine, Universidade do Estado de São Paulo, Botucatu, SP, Brazil; b Department of Pathology, Faculty of Medicine, Centro Universitário Católico Salesiano Auxilium, Araçatuba, SP, Brazil; c Department of Dermatopathology, Instituto de Patologia de Araçatuba, Araçatuba, SP, Brazil

Dear Editor,

Trichoblastomas (TB) are benign tumors derived from the hair follicle, with a low risk of malignant transformation, and are the most common neoplasm arising from nevus sebaceus of Jadassohn. They are neoplasms characterized by a biphasic differentiation into epithelium and the follicular germinative stroma. Histopathology shows a basaloid cells neoplasms in a stroma similar to follicular mesenchyme, well delimited, non-ulcerated, and restricted to the dermis, with numerous histopathological variants described.^1^

Melanotrichoblastoma is rare, with less than ten cases described in the literature; and it is different from pigmented trichoblastoma by the proliferation of intratumor melanocytes.^2^ The aim of this report is to describe a case of melanotrichoblastoma and review the literature regarding the clinical aspects of the cases described to date.

## Case report

**Figure 1:**
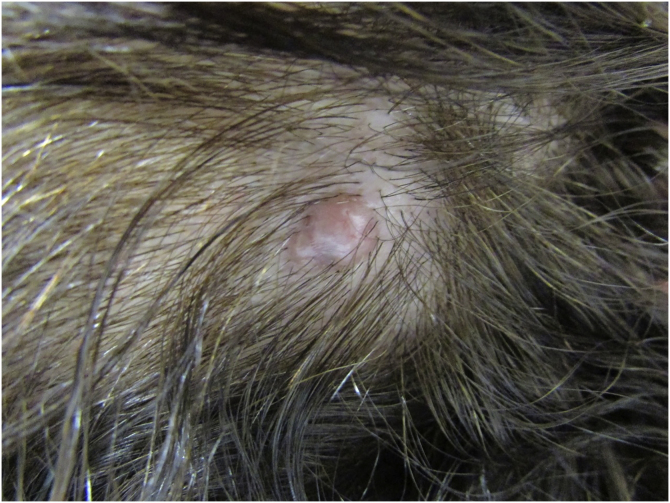
Clinical picture of the lesion, located on the scalp. A bluish spot is visible with the naked eye

**Figure 2:**
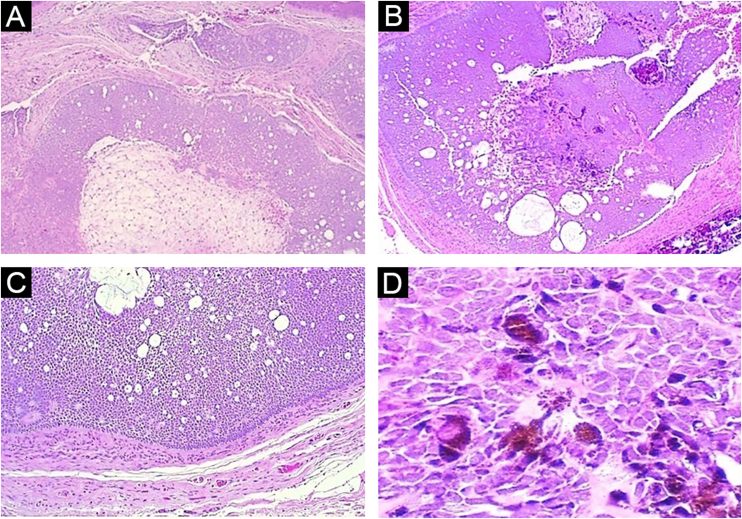
(A) Panoramic view: note the lack of connection with the epidermis and the solid-cystic component of the neoplasm (Hematoxylin & eosin, ×40). (B) Panoramic view of the deep part of the tumor, highlighting the biphasic aspect of the neoplasm and the pigmentation focus (Hematoxylin & eosin, ×40). (C) Note the peripheral palisade and cleft artifact between the neoplasm stroma and adjacent dermis (Hematoxylin & eosin, ×100). (D) Detail of basaloid epithelial cells with intense cytoplasmic melanic pigmentation (Hematoxylin & eosin, ×400)

**Figure 3:**
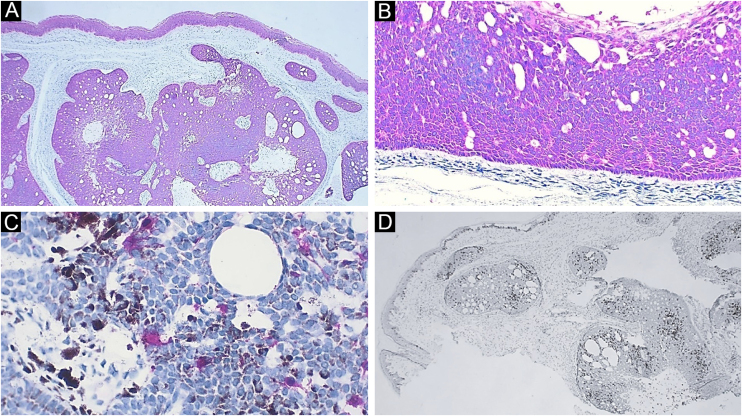
Immunohistochemistry. (A) Diffuse positivity for cytokeratin; note the epidermis as internal positive control (×40). (B) Detail of cytokeratin positivity in the neoplasm; note here also the peripheral palisade and the cleft artifact between the neoplasm stroma and the adjacent dermis (×100). (C) Detail highlighting melanocytes positive for Melan-A. Note the brownish melanin pigment in adjacent neoplastic cells (×400). (D) Ki-67 staining, demonstrating cells in the proliferative phase, with a predominantly peripheral distribution in the tumor (×40)

A 59-year-old female patient presented an asymptomatic lesion that had been growing for one month on the scalp, without the presence of associated nevus sebaceus (Fig. 1). Dermoscopy showed some arboriform telangiectasias and a single blue ovoid nest, visible to the naked eye. Lesion excision was performed due to the hypotheses of basal cell carcinoma, spiradenoma, and hidradenoma. Anatomopathological examination identified a dermal neoplasm of basaloid cells with a monotonous appearance, without connection to the epidermis, arranged in cohesive blocks, with cystic areas, and occasional peripheral palisade and multiple foci of pigmentation (Fig. 2). The epithelial cell blocks were surrounded by a hypercellular fibrous stroma showing numerous spindle cells without atypia. Cleft artifact was identified between the neoplastic stroma and the adjacent dermis. No calcification, ulceration and/or corneal cysts were observed. To quantify the melanocytes and differentiate the lesion from a pigmented trichoblastoma, the immunohistochemical study was performed (Fig. 3) using counterstaining with a magenta chromogen to avoid the influence of the melanin pigment on the interpretation of the slides. The neoplasm was positive for the cytokeratin cocktail (clone: AE1/AE3) and for Melan-A (clone A103) which highlighted the presence of numerous intratumor melanocytes. Ki-67 staining was also performed, which showed sparse positivity in cells predominantly in the periphery of the tumor.

This is the sixth case of this neoplasm described in the current literature. When analyzing the clinical data of the previously described cases (Table 1), one can observe a preferential location on the scalp, a mean age of 45 years, higher frequency in the female sex and a rare association with nevus sebaceus.

**Table 1:** Description of melanotrichoblastoma cases reported in the literature

Reference	Sex	Age (in years)	Location	Size (cm)	Lesion associated with nevus sebaceus?
Kanitakis, et al. 2002.^2^	Female	32	Scalp	2.0	No
Kim, et al. 2011.^10^	Male	51	Dorsal region	6.0	No
Hung, et al. 2012.^8^	Male	31	Scalp	1.0	Yes
Quist and DiMaio, 2017.^7^	Female	25	Scalp	1.5	No
Mizuta, et al. 2021.^9^	Female	72	Leg	11^a^	No
Present case	Female	59	Scalp	0.8	No

a This case consists in the collision of melanotrichoblastoma with seborrheic keratosis, the measure shown in the article refers to the total dimension of the lesion.

## Discussion

First described in 2002,^2^ as a distinct variant of trichoblastoma, melanotrichoblastoma still remains little known among dermatologists and pathologists, with pigmented basal cell carcinoma (BCC) as its main differential diagnosis. It is noteworthy that this differentiation becomes important due to the prognosis and follow-up.

Dermoscopy^3^ has shown that, unlike BCC, 87% of TB are symmetrical lesions, with a single homogeneous blue-gray area (in contrast to the multiple ovoid nests of BCC). A total of 61% of TB cases may have arboriform telangiectasias and 69.6% may have white structures (chrysalis included).^3^ In terms of histopathology, both are basaloid cell neoplasms, which can show different architectural patterns: large solid blocks with cystic areas, micronodules, adenoid-cystic patterns, cord-like patterns, etc. However, trichoblastomas do not have a connection with the epidermis, and exhibit a characteristic hypercellular stroma and cleft artifact between the neoplastic stroma and the adjacent dermis. BCC is usually connected to the epidermis or follicular epithelium, the stroma presents some myxoid differentiation, and there is cleft artifact between the epithelial blocks of the neoplasm and the dermis.^1^

One study evaluated BRAF, NRAS, KRAS, and HRAS gene expression, and found HRAS mutation in both a nevus sebaceus and the TB arising from it, indicating a possible common origin.^4^ There are reported cases of multiple lesions derived from nevus sebaceus, such as syringocystadenoma papilliferum, trichoblastoma, tubular apocrine adenoma, sebaceoma, follicular infundibulum tumor, and epithelioma with sebaceous differentiation.^5^ However, the neoplasm described in the present report is even more uncommon, and its difference lies in the intratumor melanocytic proliferation and not only the presence of pigment.

There is one reported case in the literature of melanoma associated with melanotrichoblastoma, called trichoblastomelanoma, in a 62-year-old female patient, with a slow-growing lesion since childhood, measuring 8 cm, with associated axillary lymph nodes.^6^ The presence of a fibrous capsule suggested degeneration of the lesion to melanoma after the TB onset, and the patient, after undergoing an extensive excision, had a two-year follow-up without disease progression.^6^ In this case, an NRAS mutation was observed, a rare mutation present in melanomas.

The present case is similar to the cases previously described in the literature, of slow-growth tumors on the scalp, in a 59-year-old woman. However, it was not associated with nevus sebaceus or seborrheic keratosis, as previously described.^2^, ^7^, ^8^, ^9^, ^10^ According to the authors review, this is the sixth reported case.

It remains under discussion whether a melanotrichoblastoma would be a variant or a synonym of pigmented trichoblastoma, since only after the advent of immunohistochemistry it became possible to identify the presence and quantify melanocytes in tumor blocks. Melanotrichoblastoma highlights the strong association of epithelial and melanocytic cells present in the structure of the hair follicle.

The interaction between keratinocytes and melanocytes in the onset of neoplastic lesions remains a little explored and a little known field, and the presence of lesions consisting of a mixture of these components indicates that it is important to better understand this relationship, even in a pathophysiological context.

## Financial support

None declared.

## Authors’ contributions

Juliana Polizel Ocanha-Xavier: Study design, investigation, follow-up and surgical excision, original manuscript drafting, review and editing.

José Cândido Caldeira Xavier-Júnior: Investigation, histopathological analysis, manuscript writing, review and editing.

## Conflicts of interest

None declared.
